# ACE2 and ACE: structure-based insights into mechanism, regulation and receptor recognition by SARS-CoV

**DOI:** 10.1042/CS20200899

**Published:** 2020-11-04

**Authors:** Lizelle Lubbe, Gyles E. Cozier, Delia Oosthuizen, K. Ravi Acharya, Edward D. Sturrock

**Affiliations:** 1 Department of Integrative Biomedical Sciences, Institute of Infectious Disease and Molecular Medicine, University of Cape Town, Observatory, Cape Town 7925, Republic of South Africa; 2 Department of Biology and Biochemistry, University of Bath, Claverton Down, Bath BA2 7AY, United Kingdom

**Keywords:** ACE inhibitor, angiotensin converting enzyme 2, COVID-19, metalloproteases

## Abstract

Angiotensin converting enzyme (ACE) is well-known for its role in blood pressure regulation via the renin–angiotensin aldosterone system (RAAS) but also functions in fertility, immunity, haematopoiesis and diseases such as obesity, fibrosis and Alzheimer’s dementia. Like ACE, the human homologue ACE2 is also involved in blood pressure regulation and cleaves a range of substrates involved in different physiological processes. Importantly, it is the functional receptor for severe acute respiratory syndrome (SARS)-coronavirus (CoV)-2 responsible for the 2020, coronavirus infectious disease 2019 (COVID-19) pandemic. Understanding the interaction between SARS-CoV-2 and ACE2 is crucial for the design of therapies to combat this disease. This review provides a comparative analysis of methodologies and findings to describe how structural biology techniques like X-ray crystallography and cryo-electron microscopy have enabled remarkable discoveries into the structure–function relationship of ACE and ACE2. This, in turn, has enabled the development of ACE inhibitors for the treatment of cardiovascular disease and candidate therapies for the treatment of COVID-19. However, despite these advances the function of ACE homologues in non-human organisms is not yet fully understood. ACE homologues have been discovered in the tissues, body fluids and venom of species from diverse lineages and are known to have important functions in fertility, envenoming and insect–host defence mechanisms. We, therefore, further highlight the need for structural insight into insect and venom ACE homologues for the potential development of novel anti-venoms and insecticides.

## Introduction

Regulatory peptides control various physiological processes ranging from fertilisation and development to immunity and nervous system function. Active peptides are formed from precursors and are degraded by peptidases after performing their functions. Two distinct but complementary peptidases at the heart of many human physiological processes are the dipeptidyl carboxypeptidase angiotensin converting enzyme (ACE) (EC 3.4.15.1), and the mono-carboxypeptidase ACE2. Both enzymes contain a characteristic HEXXH motif (where X is any amino acid) that coordinates a catalytic zinc ion and as such are members of the M2 gluzincin family of metalloproteases. ACE is well-known for its role in the renin–angiotensin aldosterone system (RAAS) where it cleaves the decapeptide angiotensin-1 (Ang I) into the potent vasoconstrictor angiotensin-2 (Ang II). Since the discovery of ACE in 1956 [1], remarkable discoveries have been made towards understanding the evolution of ACE-like proteins and their regulation, tissue distribution, structure and function which has led to the development of various classes of ACE inhibitors for the treatment of hypertension and cardiovascular disease. Despite these advances, however, the function of ACE-like proteins in many organisms remains unclear. This review provides an overview of how structural biology has improved our understanding of the function of ACE and ACE2. Moreover, it highlights the importance of continued research in this field for the potential development of novel anti-hypertensive, anti-venom and anti-viral compounds as well as insecticides.

## Biochemical properties of vertebrate ACE

In humans, transcription of a single *Ace* gene with tissue-specific promotors results in expression of two distinct isoforms, namely somatic ACE (sACE) and testicular ACE (tACE) [2]. While the tACE isoform occurs exclusively in male germinal cells [3], sACE is widely expressed and is found on the surface of endothelial, epithelial, neuroepithelial and immune cells [4]. After transcription, an N-terminal signal peptide triggers migration of the single ACE polypeptide chain to the cell membrane where it is anchored via a transmembrane region. A soluble form of sACE also exists and has been detected in blood, urine, cerebrospinal fluid and seminal fluid following cleavage of the juxtamembrane region by an as yet unidentified secretase [5]. Interestingly, the sACE polypeptide forms two homologous catalytically active extracellular domains, the N- and C-domains, each bearing an HEMGH zinc-binding motif in the active site [6] whereas the tACE isoform only forms a single domain that is essentially identical with the C-domain of sACE, except for a 36-residue serine- and threonine-rich N-terminal region present in tACE [7]. The enzymatic activity of tACE is critical for its unique function in male fertility [8]. However, Ang II is not essential for fertility as demonstrated in angiotensinogen null mice that are unable to produce Ang II [3]. tACE has also been reported to cleave glycosylphosphatidylinositol (GPI)-anchored proteins [9], but this GPIase activity seems unlikely [8,10].

While tACE is only known to be important for male fertility, sACE has a much more intricate role, and cleaves an extraordinary range of substrates through both endo- and exopeptidase action. These include Ang I, enkephalins, kinins, neurotensin, formyl-Met-Leu-Phe, substance P [4], gonadotropin-releasing hormone (GnRH), originally known as luteinising hormone-releasing hormone (LH-RH) [11], N-acetyl-Ser-Asp-Lys-Pro (AcSDKP) [12], as well as the amyloid β-peptide (Aβ) [13,14]. The high degree of homology between the sACE domains (60% overall sequence similarity and 89% active site identity) [15] suggests that they originated during an evolutionary gene duplication event and were conserved due to differences in their physiological function. Indeed, the C-domain is primarily responsible for blood pressure regulation via Ang I cleavage [6,16] whereas the peptides GnRH, AcSDKP and Aβ are preferentially cleaved by the N-domain [12–14,17,18]. The centrality of sACE in regulating the levels of these diverse peptides has made it an attractive drug target for conditions such as hypertension, kidney disease, cardiovascular disease, fibrosis and Alzheimer’s disease. The full extent of this protein’s influence on human health and disease is, however, still unknown, as was emphasised by a recent study which identified more than 240 ACE-regulated peptides in mouse plasma [19]. Even the ‘traditional’ blood pressure-regulating function of sACE is more complex than originally thought, with cross-talk occurring between the RAAS, natriuretic peptide system (NPS), kallikrein–kinin system (KKS) and the counterregulatory Mas axis of the RAAS [20].

## The human homologue, ACE2

Apart from tACE and sACE, two studies published in 2000 discovered that humans also express a homologue of ACE called ACE2 [21,22]. ACE2 (EC 3.4.17.23) is expressed in most human tissues and cell types as a type I integral membrane protein and is solubilised by the action of a disintegrin and metallopeptidase (ADAM)-17 [23]. The expression levels of ACE2 are highest in the small intestine, testis, kidneys, heart, thyroid and adipose tissue; intermediate in the lungs, colon, liver, bladder and adrenal glands; lowest in the blood, spleen, bone marrow, brain, blood vessels and muscle [24]. ACE2 contains an N-terminal peptidase domain with seven potential *N*-linked glycosylation sites that bears the characteristic HEXXH motif of M2 metalloproteases [21,22]. The ACE2 peptidase domain and the ACE domains have a 32% sequence identity and a 66% sequence similarity among all three domains, while the ACE2 peptidase domain has a 42% sequence identity (75% similarity) and a 42% sequence identity (76% similarity) with the individual N- and C-domains of ACE, respectively (Cozier et al., unpublished results). Despite these similarities, ACE2 acts as a carboxypeptidase (removing only a single amino acid from the peptide C-terminus) in contrast with the predominantly dicarboxypeptidase action of ACE. Its substrate preference also differs from that of sACE, with Ang II to Ang-(1-7) conversion being the predominant action of ACE2 [25]. Ang-(1-7) binds to the Mas receptor and forms part of the counterregulatory or protective axis of the RAAS which promotes vasodilation, decreases fibrosis and decreases inflammation by stimulation of nitric oxide synthase (NOS) [26]. Interestingly, the N-terminal peptidase domain of ACE2 is fused to a non-catalytic C-terminal domain that has 48% sequence identity to collectrin [27], a key regulator of renal amino acid transport via the transporter B^0^AT1 [28]. Unfortunately, not all ACE2 functions are beneficial. ACE2 was previously identified as the functional receptor for the severe acute respiratory syndrome (SARS) coronavirus [29] and, more recently, also for the SARS-coronavirus (CoV)-2 [30]. SARS-CoV- 2 is responsible for the coronavirus infectious disease 2019 (COVID-19) pandemic which has caused at least 23,311,719 infections and 806,410 deaths globally between December 2019 and August 2020 according to the World Health Organization. The immensity of this pandemic emphasises the importance of continued research into ACE2 and its homologues.

## ACE through the ages

**Figure 1:**
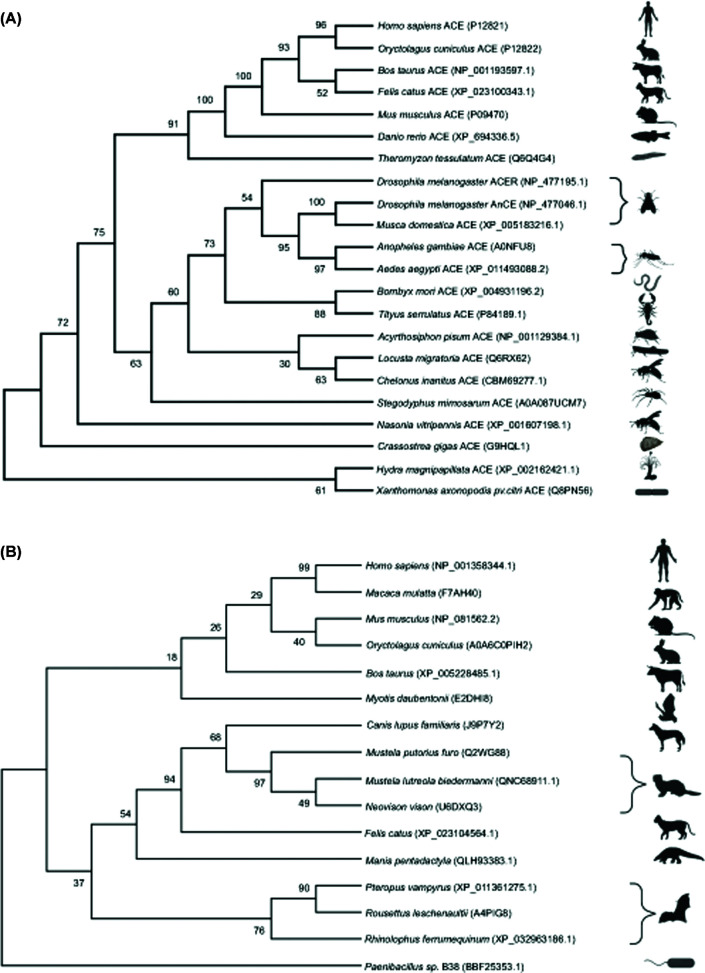
ACE and ACE2 inferred phylogeny of selected representative species Protein sequences were aligned and analysed using Maximum Likelihood (ML) with 1000 replicates in MEGA X. Accession numbers were included with each taxon and branch supports are shown at each node indicating percentage agreement of node position amongst bootstrap replicates. Branch supports below 70% are not well supported. Animal icons were obtained through BioRender. Only metallopeptidase ACE homologues were included in the analysis and only incomplete sequences for *Locusta migratoria, Chelonus inanitus* and *Myotis daubentonii* were available at the time of sequence acquisition. (**A**) Invertebrate ACE and ACE-like peptides identified in venom are clustered together and included in the representative ACE phylogeny. Moreover, vertebrate mammalian ACE has greater evolutionary support of a close relationship than seen in the invertebrate species. (**B**) ACE2 phylogeny includes sequences of pangolin and bat species indicated as potential hosts for the SARS-CoV-2 virus. In some nodes, ACE2 may be too divergent due to missing data, gene flow or recombination for higher branch support values.

Since sACE, tACE and ACE2 have such essential functions in human physiology, ACE homologues were likely preserved throughout evolution. In phylogeny, the importance of ACE and ACE2 homologues is evident from their appearance in several mammalian, insect and bacterial species (Figure 1A,B). Preservation of these homologues is particularly prominent for ACE2 when used as a SARS-CoV viral entry receptor in species such as humans, cats and bats. The housefly (*Musca domestica*) homologue was the first to be discovered and biochemically characterised [31]. Subsequently, ACE homologues have been identified in every insect genome investigated and typically share ∼40% sequence identity with vertebrate ACE. Contrary to mammals, insects have an open circulatory system and lack most other RAAS components, despite having functional ACE homologues [32]. Numerous studies have investigated the endogenous functions of insect ACE homologues and revealed their involvement in development [33], reproduction [34–36] and neuropeptide synthesis and degradation [37]. Additionally, insect ACE homologues seem to be important for modulating insect–plant interactions, as well as the innate immune system. In the swarming locust pest *Locusta migratoria*, for example, challenge with lipopolysaccharide or peptidoglycan up-regulated the expression of their ACE homologue (*Lm*ACE, *Locusta migratoria* angiotensin converting enzyme), suggesting that it is involved in defence against Gram-negative and Gram-positive bacteria [38]. Inhibition of *Lm*ACE might thus be a potential strategy to combat these locusts which are known for their devastating effect on agricultural crops worldwide. Similarly, the pea aphid (*Acyrthosiphon pisum*) homologues of ACE are important for modulating their interaction with host plants [39]. *A. pisum* ACE is constitutively expressed in the pea aphid salivary glands while ACE2 expression was induced upon feeding. Surprisingly, simultaneous knockdown of *A. pisum* ACE and ACE2 resulted in enhanced feeding and increased aphid mortality.

Although invertebrate ACE-like proteins are structurally different from mammalian sACE in that they typically consist of a single soluble domain, they have similar enzymatic activities. The study of ACE-like proteins in insects therefore not only has potential implications for food security and the development of novel insecticides [40], but also provides a convenient system to enhance our understanding of mammalian ACE function. Furthermore, the study of insect ACE homologues could potentially influence human health. The *Anopheles gambiae* and *Aedes aegypti* mosquitoes serve as vectors for the transmission of deadly diseases such as yellow fever, zika, chikungunya, malaria and dengue to human populations. Interestingly, the genome of *A. gambiae* contains nine ACE homologues of which one, *Ano*ACE9, is a two-domain protein like sACE [41]. *Staphylococcus aureus* and *Staphylococcus typhimurium* challenge of *A. gambiae* up-regulated the transcription of these *Anopheles* angiotensin converting enzyme (*Ano*ACE) homologues [42], and it was recently observed that treatment of *A. aegypti* and *A. gambiae* mosquitoes with an ACE inhibitor resulted in larval death [43]. Specific *Ano*ACE inhibitors could thus potentially be developed as novel insecticides to break the chain of malaria and arbovirus transmission.

ACE-like proteins have also been discovered in the venom of parasitic wasps [44–46], cone snails [47], spiders [48–50], scorpions [51] and jellyfish [52]. It is unclear whether these ACE-like proteins are responsible for the processing of venom peptides inside the host or whether they act after envenoming of the prey. However, envenoming often results in cardiovascular symptoms such as hypertension in addition to neurotoxic effects. Venom from the *Tityus serrulatus* scorpion could convert Ang I into Ang II and this activity was inhibited by the ACE inhibitor captopril [51]. It thus seems plausible that injection of venom containing such ACE-like proteins into the prey induces the observed hypertensive effects, but further research is required to investigate the functions of ACE-like proteins in venom.

At present, antiserum is the only specific therapy recommended by the World Health Organization for the treatment of incidents involving animal venoms. The use of antiserum, however, carries a potential risk of eliciting acute anaphylactic or delayed serum sickness reactions in some patients [53–55], and physicians are therefore hesitant to administer antiserum therapy. Compounds which selectively inhibit ACE-like venom proteins could be developed as novel alternative treatments for envenoming. On the other hand, not all animal venoms induce hypertension, with some snake [56–58], spider [59] and scorpion [60] venoms instead inducing hypotensive shock due to the presence of ACE-inhibiting bradykinin-potentiating peptides (BPPs). Further research is thus required to investigate the endogenous role of these ACE-like venom proteins and their potential as therapeutic targets.

## Structural biology of ACE-like proteins

ACE-like proteins are important for the survival of many organisms from diverse lineages and are thus attractive drug targets. The evolutionary conservation of these proteins, however, requires investigation into the species selectivity of ACE-modulating agents to avoid off-target environmental effects. Thus far, the majority of ACE homologues have been discovered using genomics approaches to study the evolution of ACE, and these proteins have therefore not been biochemically or biophysically characterised. The development of species-specific ACE-inhibitors as insecticides or antivenoms would therefore require careful comparisons of the structure and enzymatic properties of ACE homologues from these species with those of human ACE and ACE2. In this section, we provide an overview of the structures solved to date and highlight how the insights gained from human ACE and ACE2 structures enables the design of anti-hypertensive, anti-venom and anti-viral therapies.

## Overview of ACE homologue structures

The degree of sACE glycosylation (30% carbohydrates by weight) presented a major hurdle for structural studies using X-ray crystallography, since the flexibility of glycans is detrimental for crystal packing. While enzymatic deglycosylation might yield purified protein that is amenable to crystallisation, this approach is costly, has low reproducibility and potentially decreases yields which could be problematic for a technique which requires large amounts of protein. The first crystal structures of both the C- and N-domains of ACE were determined by using the glucosidase-I inhibitor *N*-butyldeoxynojirimycin (NB-DNJ) to inhibit complex oligosaccharide formation [61,62]. This approach was not suitable for high-throughput studies, however, and prompted the development of an alternative method to generate minimally glycosylated ACE proteins. Systematic mutation of *N*-linked asparagines to glutamines revealed the minimum glycan requirement for correct folding, localisation and stability of the ACE domains, and enabled crystallisation of minimally glycosylated active C- and N-domains’ proteins [63,64]. To date, this has been expanded to 50 examples of the two domains, with both native structures and numerous examples in complex with substrates, products or inhibitors [65–69]. Structures of the human ACE domains provided detailed insight into the mechanisms of substrate hydrolysis [68,70,71], and enabled the design of domain-selective ACE inhibitors for the specific treatment of hypertension (C-domain) [72–74] and fibrosis (N-domain) [67,75,76].

Many ACE ligands have also been co-crystallised with the *Drosophila melanogaster* AnCE (*Drosophila* angiotensin converting enzyme) homologue, highlighting key differences in the active sites of ACE and AnCE [77–82]. Recently, the structure of *A. gambiae Ano*ACE2 complexed to an ACE inhibitor was also solved, and active site comparisons revealed potential avenues for the development of *Ano*ACE2-specific inhibitors for use as insecticides [83]. In 2004, the first crystal structures of human ACE2 were solved in apo- and inhibitor-bound forms in response to the SARS coronavirus epidemic [84]. After the epidemic ended, research into ACE2 waned but saw a rapid surge this year with the emergence of SARS-CoV-2 in December 2019. To date, numerous crystal or cryo-EM structures have been solved of full-length ACE2 or the peptidase domain in complex with the coronavirus spike proteins, small molecule inhibitors or the amino acid transporter B^0^AT1 [84–90]. The range of structures available for both ACE and ACE2 enables detailed comparisons of their overall structures, active sites and the structural changes that occur during opening and closing of the peptidase domains.

## Global comparison of the ACE and ACE2 crystal structures

**Figure 2:**
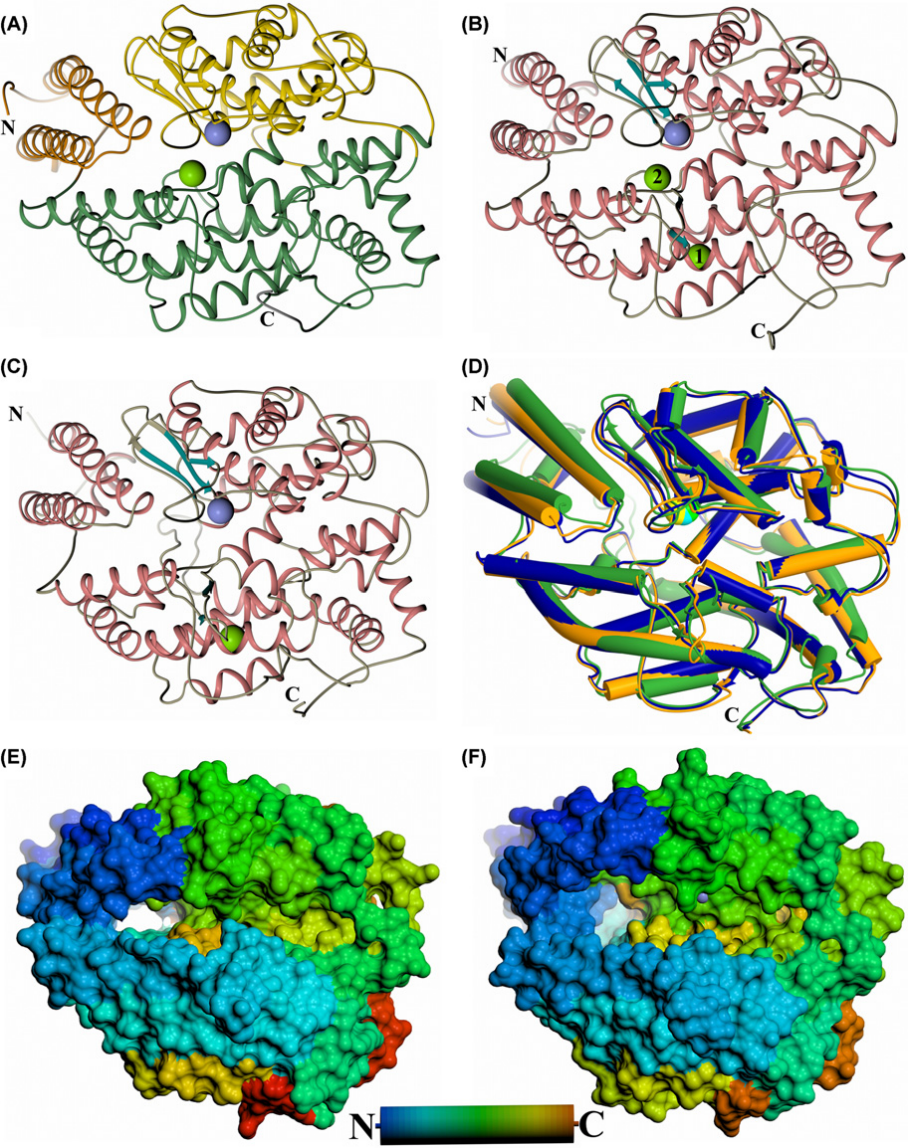
Schematic structure representations Crystal structures of (**A**) typical closed ACE N-domain (PDB code: 6F9V), (**B**) typical closed ACE C-domain (PDB code: 6H5W), (**C**) closed ACE2 (PDB code: 1R4L). (**D**) Overlay of ACE N- and C-domains with ACE2 ACE domain (blue, orange and green, respectively, with active site zinc ions shown as lighter coloured spheres). (**E,F**) Surface view of the ACE N-domain (PDB code: 6ZPQ) and ACE2 open structures (PDB code: 1R42), respectively. (A) shows subdomain 1 and 2 in orange (the lid-like region in dark orange) and green colour, respectively. The chloride and active site zinc ions are depicted as green and lilac spheres, respectively. For clearer comparison panels (B,C) have been coloured by secondary structure with α-helices and β-strands coloured rose and dark cyan, respectively.

The first crystal structures of the ACE2 peptidase domain were reported as an open native structure and a closed conformation in complex with the inhibitor MLN-4760 [84]. In contrast, the majority of ACE structures solved to date have shown the domains in a closed conformation with the open conformation only recently elucidated [91]. Crystal structures of the ACE and ACE2 peptidase domains in the closed conformation (Figure 2A–C) indicate that the overall fold of these domains is retained [65,66,84], in line with the high degree of sequence conservation between them. All three structures show an ellipsoid shape that is largely α-helical, with all helices and β-strands being conserved in each peptidase domain. An overlay of these structures (Figure 2D) shows that the overall arrangement of these secondary structure features is also well conserved, with ACE2 showing a marginally closer similarity to the ACE C-domain (root mean square deviation (RMSD) 1.92 Å for 578 Cα atoms) than the N-domain (RMSD 2.42 Å for 570 Cα atoms) (Cozier et al., unpublished results). The majority of differences between these structures are in the loop regions, in particular between α-helices 7 and 8, which is 10 residues longer in ACE2.

The tertiary structure of ACE shows the ellipsoid, divided into two approximately equal-sized subdomains (1 and 2) (Figure 2A). The subdomains are not simply the N- and C-terminal halves of the protein, instead the polypeptide chain crosses between the two subdomains five times (subdomain 1: ACE2 19–102, 286–431 and 521–580, N-domain 11–97, 272–417 and 507–566, and C-domain 38–121, 294–439 and 529–588; subdomain 2: ACE2 103–285, 432–520 and 581–612, N-domain 98–271, 418–506 and 567–601, and C-domain 122–293, 440–528 and 589–623) at the base of the ellipsoid. The open structures of the ACE N-domain [91] and ACE2 [84] retain the same secondary structure elements and show that the two subdomains open in a clam-shell like manner for both enzymes, resulting in a deep groove leading to the active site at the base (Figure 2E,F). In the closed structures, the two subdomains are tightly connected allowing limited access into the active site and the deep groove is replaced by a two-lobed binding cavity with the active site zinc ion coordinated at the narrowest point (Figure 2B,C). Comparison of the two open structures shows that ACE2 has opened further than observed for ACE N-domain (maximum opening reported as 10.4 Å for ACE N-domain and ∼15 Å for ACE2). It is likely that the ACE N-domain structure has crystallised in a partially open conformation that may be stabilised by a series of N-domain residues located at the subdomain interface distal from the binding site which affect binding affinity when mutated [92,93]. It can be envisaged that the deep groove of the open structure only allows elongated, linear peptides access to the active site, preventing cleavage of folded proteins.

The first approximately 100 residues of the ACE domains comprise largely of two long helices that form a highly flexible region of subdomain 1 that has been termed a ‘lid-like’ domain (Figure 2A) [61]. Not only does it open with the rest of subdomain 1 in the clamshell-like manner but the other end of these long helices also pivot towards and away from subdomain 2. This is usually most apparent in one molecule of the N-domain crystal structure asymmetric unit, in particular it was possible to model two conformations of this region in the structure in complex with the ACE inhibitor sampatrilat [66]. The base of this ‘lid-like’ region is adjacent to a hole that leads into the non-prime lobe of the binding cavity. This may have implications for the ability of these ACE domains to cleave peptides that are too long to fit into the binding cavity, which is described in more detail below [91].

## Post-translational modifications

The peptidase domains of both ACE and ACE2 are heavily glycosylated. The N-domain of ACE is glycosylated at nine of the ten predicted *N*-linked sites (asparagines 9, 25, 45, 82, 117, 131, 289, 416 and 480), the C-domain of ACE is glycosylated at six of the seven predicted *N*-linked sites (asparagines 72, 90, 109, 155, 337 and 586), and the ACE2 peptidase domain is glycosylated at all six predicted *N*-linked sites (asparagines 53, 90, 103, 322, 432 and 546) [64,84,94]. Removing these glycans through systematic mutation of the *N*-linked asparagines to glutamines has determined that some of these glycosylation sites are required for correct folding, localisation and stability of the ACE domains [63,64]. The minimally glycosylated active enzymes used for crystallisation retained the N-domain sites Asn^45^, Asn^416^ and Asn^480^ and the C-domain sites Asn^72^ and Asn^109^ while the remaining non-essential asparagines were mutated to glutamine residues [63,64]. It is unclear what the precise functions of these glycosylation sites are, but two are conserved in all three peptidase domains (ACE N-domain Asn^45^ and Asn^82^; ACE C-domain Asn^72^ and Asn^109^; ACE2 Asn^53^ and Asn^90^). Only Asn^45^ is required for ACE N-domain folding, processing and thermal stability, but both sites are needed for the ACE C-domain. In addition, N-domain Asn^131^ and C-domain Asn^155^ are on a short loop between the β1 and β2 strands and, while this loop is two residues longer in the C-domain, this glycosylation site may have similar functions in both domains. Likewise, while N-domain Asn^416^ and ACE2 Asn^432^ are not equivalent residues in the sequences, they are both on the same loop, albeit this loop is one residue longer in ACE2. This loop has been implicated in the opening mechanism of these domains (see below), indicating that their glycosylation may be involved, although this site is not conserved in the ACE C-domain.

## Chloride ion regulation

ACE C-domain binds two chloride ions (Cl1 and Cl2) (Figure 2B) [61]. Cl1 is located 20.7 Å from the active site zinc ion and is bound by Arg^186^, Trp^485^, Arg^489^ and a water molecule. Cl2 is closer to the zinc (10.4 Å) than Cl1, and is coordinated to Arg^522^, Tyr^224^ and a water molecule. ACE N-domain binds a single chloride ion with only the Cl2 site conserved (Arg^500^ and Tyr^202^) (Figure 2A) [62]. In contrast, while ACE2 also binds a single chloride ion, this is bound in the equivalent Cl1 site of ACE C-domain interacting with ACE2 residues Arg^169^, Trp^477^ and Lys^481^ (Figure 2C) [84]. The activity of both domains of ACE is dependent on chloride ions, with the C-domain to a greater extent, and the degree of dependence is affected by the specific substrate involved [95–98]. The Cl1 site was shown to only have a limited effect on activity, probably by structurally stabilising the S′ subsites. In contrast, the Cl2 site which is conserved in both ACE domains has greater importance in controlling activity by stabilising the substrate in the active site [99]. The type of substrate may control the access of chloride ions into the Cl2 site, thereby explaining substrate-specific dependence on chloride ions. It is interesting that ACE2, even though it only has the Cl1 site conserved, is also dependent on chloride ions for activation [100]. One explanation is that ACE2 has a second unique chloride binding site when bound to substrate or inhibitor, but this binding site was not observed in the MLN-4760 complex structure due to the low 3.0 Å resolution [84]. Another explanation could be that with ACE2 being a monopeptidase, the more subtle stabilisation by the Cl1 site has a greater effect when only an S1′ subsite is occupied.

## Active site comparisons

Both ACE and ACE2 are members of the zinc metalloprotease family and contain the HEXXH + E (downstream glutamate residue) zinc binding motif. As described above, this zinc binding site is located at the base of a deep cleft in the open structure, which rearranges to be at the junction of a two-lobed cavity in the closed structure. The non-prime lobe in both enzymes is larger than the prime lobe allowing space for the longer N-terminal residues of the peptide substrates. Comparison of the active site in the closed structures gives insights into the structural differences that result in ACE2 being a carboxypeptidase, whereas ACE is predominantly a dipeptidyl carboxypeptidase, but also has other exo- and endopeptidase activities.

**Figure 3:**
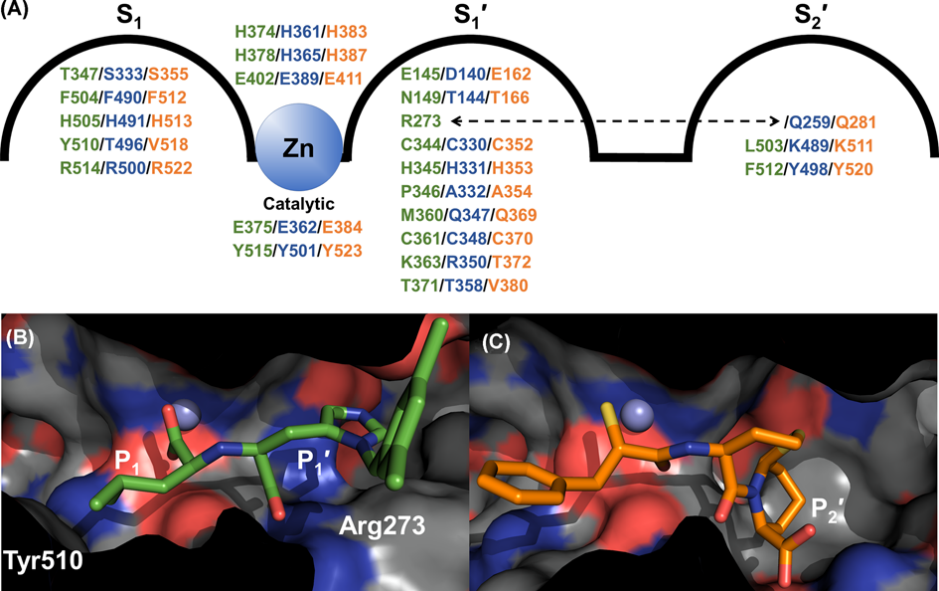
Variation in subsites between ACE and ACE2 Panel (**A**) depicts the relevant residues in the S_2_′-S_1_ subsites with ACE2, N- and C-domains coloured green, blue and orange, respectively. The dotted arrow indicates that ACE2 S_1_′ Arg^273^ is equivalent to an S_2_′ residue in ACE. Binding cavity of (**B**) ACE2 in complex with MLN-4760 (PDB code: 1R4L) and (**C**) ACE C-domain in complex with omapatrilat (PDB code: 6H5W) showing variation in available space within the S_2_′-S_1_ subsites.

The active sites (S_2_′-S_2_ subsites) of the N- and C-domains of ACE are highly conserved with 90% identity, although these domains have distinct substrate specificities and inhibitor affinities even for ligands that bind only in these subsites. A comparison of 21 residues that are within 4.5 Å of the bound MLN-4760 in the ACE2 complex with the equivalent residues in the ACE domains (ACE2 residues written in text, equivalent ACE domain residues included in Figure 3A) showed the zinc binding and catalytic glutamate residues are conserved in all three domains (His^374^, Glu^375^, His^378^ and Glu^402^) as are seven other residues (Cys^344^, His^345^, Cys^361^, Phe^504^, His^505^, Arg^514^ and Tyr^515^) (updated from ACE C-domain/ACE2 comparison in [84] to include ACE N-domain as well). A further three residues are conserved between ACE2 and one of the ACE domains (Glu^145^, Asp^368^ and Thr^371^), and the remaining seven are not conserved between ACE2 and ACE (Asn^149^, Arg^273^, Pro^346^, Thr^347^, Met^360^, Lys^363^ and Tyr^510^), although most of these are conserved between the two ACE domains. The non-conserved residues are likely to contribute to the differences in substrate specificity, reaction type and inhibitor affinity seen between ACE and ACE2. However, as has been shown with ACE, there is less conservation of residues in the subsites beyond S_2_, and some residues that are distal from the prime binding site are also not conserved but still have an effect on the affinity of some ACE inhibitors.

Multiple structures of both ACE domains have shown that the carboxy terminus of peptide ligands bind strongly to conserved residues in the S_2_′ subsite (N-domain/C-domain; Gln^259^, Lys^489^ and Tyr^498^; Gln^281^, Lys^511^ and Tyr^520^). These interactions are important for ligand binding in ACE, as to date every native structure reported has a ligand scavenged from the expression, purification or crystallisation environment bound to this region [61,62,101]. With this being the S_2_′ subsite, it will be part of the mechanism for positioning and orientating the peptide substrate for dipeptidyl carboxypeptidase activity. In contrast, none of these residues are conserved in ACE2 (Arg^273^, Leu^503^ and Phe^512^, respectively) (Figure 3A), and these changes explain why ACE2 is a carboxypeptidase, cleaving only a single residue off the C-terminus of peptides. Not only does ACE2 lack a P_2_′ carboxy terminus binding site, but the bulky Arg^273^ sidechain is orientated such that it blocks the S_2_′ subsite (Figure 3B,C) and instead coordinates with the carboxy terminus of the P_1_′ residue from the ligand [84]. Even though residues are not conserved, the S1′ subsite is somewhat similar between the ACE and ACE2 domains in that it favours either hydrophobic residues (due to ACE2/N-domain/C-domain residues Pro^346^/Ala^332^/Ala^354^ and Thr^371^/Thr^358^/Val^380^), or more extended groups such as seen with MLN-4760 and the lysine group of the ACE inhibitor lisinopril [61,62,84]. These extended groups access a region that is poorly conserved between the ACE domains, and so could also lead to the discovery of domain-specific inhibitors. The S_1_ subsite of ACE2 has been shown to only accommodate small- and medium-sized side chains, due to the space available between Thr^347^ and Tyr^510^ [84,100]. In contrast, this subsite in both the N- and C-domains is larger with residues Ser^333^/Thr^496^, and Ser^355^/Val^518^, respectively (Figure 3B,C). The smaller S_1_ subsite in ACE2 explains why bradykinin (RPPGFSPFR) is not a substrate because there is not enough space for the penultimate (for a carboxypeptidase) phenylalanine residue.

Both ACE and ACE2 cleave a variety of substrates in addition to their most well-known substrates: Ang I (ACE), Ang II (ACE2) and bradykinin (ACE). The majority of these are 12 residues or less and peptides of this length can be accommodated within the binding cavity of the closed structure. This was demonstrated by the structures of both N- and C-domain ACE structures in complex with BPPb, where the ligand extends across the full length of the non-prime lobe [69,102]. However, both ACE and ACE2 are capable of cleaving much longer substrates such as amyloid-β peptides (ACE) and apelin-36 (ACE2), both of which are over 35 residues in length [13,14,100]. It has been unclear how these could be accommodated and cleaved by these domains, but a recent native C-domain structure has shed light on a possible explanation [91]. A small hole in the non-prime lobe adjacent to the flexible ‘lid-like’ region has multiple conformations in the N-domain structures thereby altering the size of the hole. Both domains of ACE and the ACE2 peptidase domain have conserved ‘lid-like’ regions and possess this hole. In the recent C-domain structure [91], the C-terminus of a symmetry related cACE molecule from the crystal lattice is shown to insert through this hole, extend across the non-prime lobe, and coordinate with the active site zinc ion. Therefore, it was suggested that long peptides could be accommodated by the N-terminal portion passing out through this hole and occupy space outside the ACE domain. In addition, it is possible that this hole could provide an exit point for the N-terminal products of peptidase activity. Further work is required to confirm these suggestions.

## Hinge mechanism of ACE2 and ACE

With the first ACE2 crystal structures being reported as the native open conformation and the closed inhibitor bound complex, it was immediately apparent how this peptidase domain enabled substrate entry to the active site. In contrast, with the N- and C-domains of ACE only crystallising in the closed conformation until recently, the opening of ACE could only be predicted using molecular modelling and comparisons with ACE2. There have been several of these molecular modelling studies using normal mode analysis and molecular dynamics examining not only the domain opening directly, but also the effect of site-directed mutations on this movement [92,101,103]. With the recent crystal structure of ACE N-domain in the open conformation [91], and re-examination of the earlier C-domain structure in complex with BPPb that was shown to have opened slightly [69], it can be confirmed that all these domains open with the same mechanism. The ACE N-domain structure did not show as large a degree of opening as ACE2, and it was concluded that the N-domain had crystallised in a partially open conformation, possibly stabilised by residues at the subdomain interface coordinating magnesium ions from the crystallisation conditions. One of the molecular dynamics studies using an ACE C-domain structure with the co-crystallised Ang II ligand removed, showed that during a long 400 ns simulation the C-domain went through various states of closed, partially open, and fully open conformations that were similar to those of the ACE2 structures [103].

**Figure 4:**
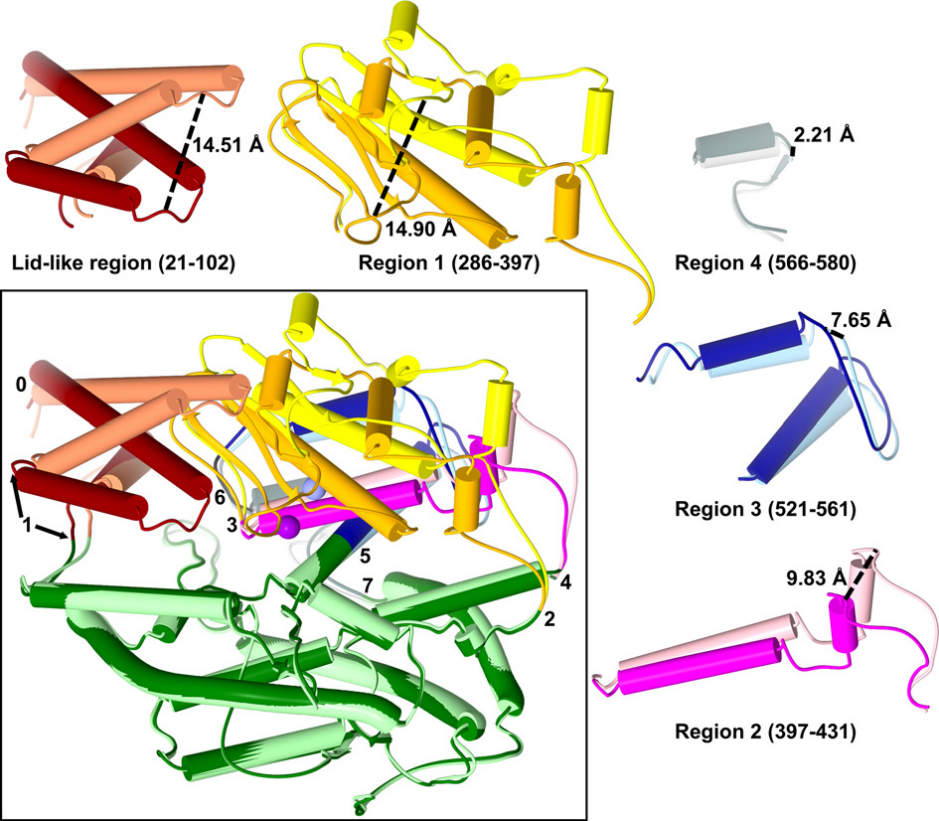
Mechanism of ACE domain opening Overlay of the open and closed ACE2 structures highlighting the regions that move during the domain opening with the hinges about which the regions pivot numbered. Each moving region is also shown in isolation for clarity with the largest movement point marked. Subdomain 2 is shown in dark green/light green, with the moving regions of subdomain 1 coloured dark red/coral (residues 21–102), orange/yellow (residues 286–397), magenta/light pink (residues 397–431), dark blue/light blue (residues 521–561) and dark grey/light grey (residues 566–580). Zinc ion is shown as violet/lilac sphere. Darker colours are the closed structure.

In essence, this opening is the movement of the two subdomains away from each other in a clamshell-like manner. This is possible because all the regions of the polypeptide that cross between the two subdomains are located at the base of the ellipsoid and act as hinge regions or pivot points (Figure 4). However, the opening is more complicated with further hinge regions located within subdomain 1 to allow for the movement of secondary structure sections required for large conformational changes during opening and closing. A total of seven hinge regions were identified from the crystal structures, some of which are single residue pivot points, while others are a short section of residues. Subdomain 1 can be split up into five sections between these hinge regions, each with their own degree of movement which correlates to their proximity to the base or clamshell lip. The ‘lid-like’ region (residues 21–102; ACE2 numbering is used throughout this description) is the first of these sections and, as described above, it has a more complicated variation in movement than the rest of subdomain 1. The domain opening part of its movement is about hinges 0 (residue 24) and 1 (residues 83–105), which are located at the base of the two long helices.

The remainder of subdomain 1 can be split into four sections (residues 286–397, 397–431, 521–561 and 566–580 for sections 1–4, respectively) that are approximately stacked on top of each other from the base to clam-shell lip (Figure 4). The amount each section pivots correlates with its position on the clam-shell, with those at the base moving less than those near the lip. Section 4, at the base of the clam-shell, pivots about hinges 6 (residues 562–565) and 7 (residue 581), and moves the least of all the sections; it is crucial for allowing the conformational changes in the rest of subdomain 1 above it. Directly above is section 3, which also pivots about hinge 6, as well as hinge 5 (residue 521). Section 2 contains the zinc binding glutamate residue (Glu^402^) and pivots about hinges 3 (residue 397) and 4 (residue 431). Hinge 4 is on the loop that contains a glycosylation site in both the ACE N-domain and ACE2, but not in the C-domain of ACE. The ACE N-domain and ACE2 peptidase domain are similar in being both N-terminal, with other domains (the C-domain and collectrin-like domain, respectively) located between them and the membrane. In contrast, the ACE C-domain is sandwiched between the N-domain and the membrane. Therefore, it is interesting to speculate that glycosylation may play some role in controlling the opening mechanism for N-terminal peptidase domains. Finally, subdomain 1 not only contains the HEXXH motif (zinc binding histidines and catalytic glutamate), but also forms the lip of the clam-shell. It shows the largest degree of movement between the open and closed conformations, shares hinge 3 with subdomain 2, and also pivots about hinge 2 (residue 286).

## ACE2 and SARS-CoV-2 crystal structure

Coronaviruses have evolved to use many different cell surface proteins as receptors for infection [104], with SARS-CoV (2002/3 epidemic), NL63-CoV (common cold) and SARS-CoV-2 (2019/2020 pandemic) all utilising ACE2 as their receptor [29,105–110]. It is an interaction between the receptor-binding domain (RBD) of the coronavirus cell surface spike protein with the ACE2 receptor that facilitates infection. To understand how the spike protein on the surface of coronaviruses exploits ACE2, it is important to examine the molecular basis of the interaction.

**Figure 5:**
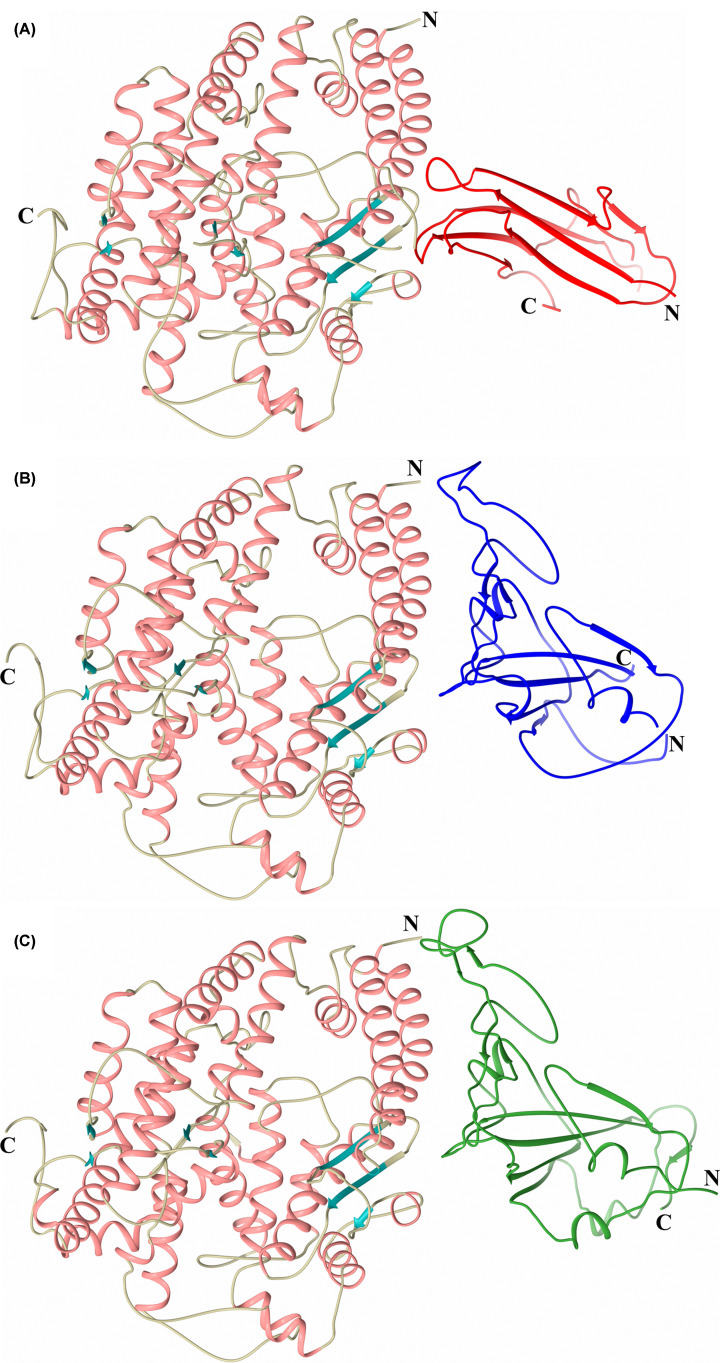
ACE2 structures in complex with coronavirus RBDs Schematic representations of ACE2 in complex with RBDs from (**A**) NL63-CoV (PDB code: 3KBH), (**B**) SARS-CoV (PDB code: 2AJF) and (**C**) SARS-CoV-2 (PDB code: 6M0J). ACE2 domains are coloured by secondary structure with α-helices and β-strands coloured rose and dark cyan, respectively. NL63-CoV, SARS-CoV and SARS-CoV-2 RBDs are coloured red, blue and green, respectively.

The first observation of a coronavirus spike protein interacting with ACE2 was the crystal structure of the SARS-CoV RBD in complex with the ACE2 peptidase domain (Figure 5B) [86]. There are two copies of the ACE2/SARS-CoV heterodimer in the asymmetric unit, with one copy showing ACE2 in an equivalent fully open conformation as that observed in the crystal structure of ACE2 alone. The second copy shows ACE2 in a slightly more closed conformation, reminiscent of that seen for the ACE N-domain open structure (RMSD 1.45 Å for 560 Cα atoms). Some studies have suggested that ACE2 inhibitors decrease the binding of the SARS-CoV spike protein to ACE2 and prevent viral entry into the host cells [111]. However, this seems unlikely as interaction of the spike protein RBD to inhibitor-bound ACE2 abrogates the inhibitory effect due to dissociation of the inhibitor [112]. The two ACE2 conformations observed in the SARS-CoV RBD complex structure further suggests that the interaction of the SARS-CoV spike protein is unaltered by the opening and closing of ACE2.

The SARS-CoV RBD forms a concave surface which interacts with the convex first long helix (residues 21–53) and loop residues 82–83 at the end of the second long helix of the ACE2 ‘lid- like’ region, as well as a β-hairpin loop (residues 352–354) and an α-helix loop (residues 325–330) also of ACE2 subdomain 1 (Figure 5B). In addition, while no strong interactions are observed, the glycosylation chain on Asn^90^ of ACE2 points towards the SARS-CoV RBD with the β-D-mannose modelled 4.0 Å away (examination of the experimentally determined electron density maps indicates it could be even closer than this). This glycosylation site is one of the two conserved in both ACE domains, so while it cannot explain SARS-CoV specificity for ACE2 over ACE, it still may have some effect on interaction between ACE2 and SARS-CoV. Indeed, mutation of ACE2 Asn^90^ to remove the glycosylation site increases the rate of infection by pseudotyped lentiviruses by enhancing spike protein-mediated binding [86,113].

Variations in human, rat and mouse ACE2 sequences have an effect on susceptibility to SARS-CoV [114]. Rat ACE2 has a glycosylated asparagine in place of the Met^82^ of human ACE2, which would not only interfere with the interactions from this loop with the SARS-CoV RBD but would also cause steric hindrance. This is likely to be the main reason why rat ACE2 does not support infection by SARS-CoV. A histidine has also replaced Lys^353^ of human ACE2 in rats and mice. The human Lys^353^ is on the β-hairpin loop and has a critical interaction with Thr^487^ of SARS-CoV RBD. This is consistent with mouse infection by SARS-CoV via ACE2 being inefficient, and mutation of this mouse histidine residue to the human equivalent lysine residue results in high-level infection by SARS-CoV [114]. It is interesting to note that this β-hairpin loop in ACE is one residue longer in both N- and C-domains. While there is a lysine residue on this loop (Lys^341^ and Lys^353^ for the N- and C-domain, respectively), it does not occupy the same spatial position to allow for the interactions observed in the ACE2/SARS-CoV complex. In addition, an overlay of ACE domains with ACE2 shows that the ACE lysine would in fact cause steric hindrance with a loop (residues 485–491) from the SARS-CoV RBD. Therefore, this β-hairpin loop region maybe a significant part of SARS-CoV’s specificity for ACE2 over ACE.

A second crystal structure of ACE2 in complex with a coronavirus RBD was reported using the spike protein from NL63-CoV (Figure 5A) [87]. NL63-CoV is the only group I coronavirus that is known to use ACE2 as its receptor [105], but it was unclear from sequence comparisons with SARS-CoV how this interaction occurred. The ACE2/NL63-CoV complex structure showed that its RBD is structurally very different from that of the SARS-CoV RBD. It does not interact over an extensive area of ACE2 as observed in the SARS-CoV complex, but instead is focused on the area near the end of the first long helix of the ACE2 ‘lid-like’ domain. In this region, it shares similarities with the SARS-CoV RBD in that it not only interacts with helix residues 30-41, but also the β-hairpin loop (residues 353-356) and the other α-helix loop (residues 321-330). In addition, as observed in the ACE2/SARS-CoV complex, Lys353 of ACE2 plays a crucial role in the interaction with the NL63-CoV RBD, where its alkyl chain is predominantly at the centre of hydrophobic patches on both ACE2 and NL63- CoV RBD. An overlay of ACE peptidase domains onto the ACE2/NL63-CoV structure shows that the ACE lysine residue (Lys341 and Lys353 for N- and C-domain, respectively) on the equivalent β-hairpin loop that contains the crucial ACE2 Lys353 would cause extensive steric hindrance with the NL63-CoV RBD.

As the scale of the 2020 SARS-CoV-2 pandemic widened, it became increasingly important to understand the interaction of its spike protein with its receptor ACE2, and subsequently there have been several 3D structures published using a variety of approaches. A method using neutral amino acid transporter B^0^AT1 to stabilise the full length ACE2 enzyme (including the transmembrane helix) was utilised to give a cryo-EM structure of the SARS-CoV-2 RBD/ACE2/B^0^AT1 complex [85]. The dimer of heterodimers seen in the ACE2-B^0^AT1 complex left the SARS-CoV-2 RBD binding region of ACE2 unobstructed, and the resulting interaction had a local 3.5 Å resolution. The RBD contains a core and a receptor-binding motif (RBM) of which the latter mediates direct contact with ACE2. A chimeric protein using the RBD core from the SARS-CoV spike protein with the RBM region of SARS-CoV-2 (an arginine residue from SARS-CoV RBM that is not conserved in SARS-CoV-2 was retained in the chimera to aid crystallisation) was used to obtain a 2.68 Å crystal structure in complex with the ACE2 peptidase domain [115]. Two crystal structures at 2.45 and 2.5 Å have now been reported of the native SARS-CoV-2 RBD in complex with the ACE2 peptidase domain (Figure 5C) [89,116]. While all these structures agree essentially, the higher resolution structures using native SARS-CoV-2 RBD allow for more detailed, unbiased examination of the protein- protein interface.

A comparison of the ACE2/SARS-CoV-2 RBD complex with that of ACE2/SARS-CoV RBD shows that the ACE2 peptidase domains adopt very similar open conformations (using the heterodimer molecule with the fully open peptidase domain in the ACE2/SARS-CoV RBD complex) with an RMSD of 0.52 Å for 595 Cα atoms. The overall structure of the SARS-CoV-2 RBD is similar to that of the SARS-CoV RBD (Figure 5B,C) in the two structure complexes (RMSD of 1.2 Å for 174 aligned Cα atoms), and the less well-conserved RBM region is also structurally well conserved (RMSD of 1.3 Å for 69 Cα atoms) with only one distal loop showing obvious variation. There are 14 positions in the extended RBM that have interactions with ACE2 in both complex structures (Table 1), with 8 identical and 5 similar. While SARS-CoV-2 Gln^498^ and SARS-CoV Tyr^484^ of the last position are not similar, they both share equivalent interactions with ACE2 (Asp^38^, Tyr^41^, Gln^42^, Leu^45^ and Lys^353^). Of the five similar positions mentioned, Leu^455^/Tyr^442^ and Asn^501^/Thr^487^ positions both have similar interactions in both complexes (with ACE2 residues Asp^30^, Lys^31^ and His^34^, and Tyr^41^, Lys^353^, Gly^354^ and Asp^355^, respectively). The other three positions show differences in the two structures, and in each case, there are more interactions observed in the ACE2/SARS-CoV-2 complex than in the ACE2/SARS-CoV structure. Phe^456^ of SARS-CoV-2 has interactions with Thr^27^, Asp^30^ and Lys^31^ of ACE2, with Leu^443^ of SARS-CoV limited to interacting with Thr^27^ of ACE2. Phe486 of SARS-CoV-2 interacts with Gln^24^, Leu^79^, Met^82^ and Tyr^83^ of ACE2, compared with only Leu^79^ and Met^82^ of ACE2 interacting with Leu^472^ of SARS-CoV. Gln^493^ of SARS-CoV-2 interacts with Lys^31^, His^34^ and Glu^35^ (including a hydrogen bond) of ACE2, whereas Asn^479^ of SARS-CoV interacts with a single residue (His^34^) of ACE2. Lys^417^ of SARS-CoV-2 uniquely interacts with Asp^30^ of ACE2, where the equivalent residue of SARS-CoV (Val^404^) does not interact with ACE2. Conversely Gln^325^ and Glu^329^ of ACE2 only interact with SARS-CoV (Arg^426^ and Ile^489^, and Arg^426^, respectively). Similar to the structures of ACE2 in complex with SARS-CoV and NL63-CoV RBDs, Lys^353^ of ACE2 is critically important in binding the SARS-CoV-2 RBD.

**Table 1:** RBM interacting residues

SARS-CoV-2	SARS-CoV
Conserved	
Tyr^449^	Tyr^436^
Tyr^453^	Tyr^440^
Asn^487^	Asn^473^
Tyr^489^	Tyr^475^
Gly^496^	Gly^482^
Thr^500^	Thr^486^
Gly^502^	Gly^488^
Tyr^505^	Tyr^491^
Similar	
Leu^455^	Tyr^442^
Phe^456^	Leu^443^
Phe^486^	Leu^472^
Gln^493^	Asn^479^
Asn^501^	Thr^487^
Unconserved	
Gln^498^	Tyr^484^

These similarities and differences combined mean that overall, there are more interactions between ACE2 and SARS-CoV-2 (288 in total of which 16 are hydrogen bonds) than observed between ACE2 and SARS-CoV (213 in total of which 11 are hydrogen bonds). This is consistent with the observations that SARS-CoV-2 has a higher binding affinity than SARS-CoV for ACE2 [89,90,116].

## Molecular basis for therapeutic interventions

### ACE2 inhibitors

ACE2 and its product Ang-(1-7) have important protective effects in the heart, kidney, central nervous system and blood vessels. Thus, ACE2 has been posited as a potential therapeutic target for cardiovascular disease, driving the need for the investigation of the structure of ACE2.

The first class of ACE2 inhibitors was developed by Millennium Pharmaceuticals in an effort to understand its biological importance and potential role in disease [117]. They used a rational design approach together with substrate screening to develop a library of non-hydrolysable His-Leu mimetics that occupied both the S_1_ and S_1_′ subsites of ACE2, containing a carboxylate zinc-binding group. Inhibitor potency and selectivity was optimised by probing the P_2_, P_1_ and P_1_′ sites. A series of compounds with 3,5 disubstituted benzyl P_2_ groups inhibited ACE2 in the picomolar range with excellent selectivity and compound 16, with a 3,5-dichlorobenzyl P_2_ group (also known as MLN-4760), showed the greatest potency. Surprisingly, the crystal structure of ACE2 in complex with MLN-4760 revealed that the inhibitor binds in the opposite orientation with the isobutyl group interacting with the S_1_ subsite [84]. The 3,5-dichlorobenzylimidazole group binds in the larger S_1_′ site formed by the channel between the two subdomains of ACE2 (Figure 3B). The S_1_′ residues Phe^274^, Pro^346^, Thr^371^ and Met^360^ and the Cys^344–361^ disulphide bond create a hydrophobic environment, and Asn^149^, Glu^145^, Cys^344^, Cys^361^, Met^360^ and Asp^368^ interact with the two chlorides. The isobutyl carboxylate binds to the zinc, which is also coordinated by His^374^, His^378^ and Glu^402^. The substitution of the ACE2 S_2_′ Arg^273^ for a glutamate in ACE provides the molecular basis for substrate and inhibitor selectivity and explains why ACE2 is not inhibited by classical ACE inhibitors that occupy the S_1_′ and S_2_′ subsites.

The crystal structure of ACE2 in complex with MLN-4760 heralded a structure-based pharmacophore modelling approach and virtual screening for new ACE2 inhibitors [118]. A total of 3.8 million compounds from various commercial databases were searched and 17 compounds were selected and validated with a fluorogenic ACE2 activity assay using the synthetic ACE2 substrate Mca-APK(Dnp). The most promising compound, 4S-16659, exhibited an *IC_50_* value of 62 µM. Interestingly, the docked structure, which aligned well with MLN-4760, suggested a novel 1,2,4-triazole heterocyclic zinc-binding group on 4S-16659.

Complete inhibition of ACE2 *in vivo* required relatively high doses of MLN-4760 indicating a low bioavailability for this nonpeptidic inhibitor [119]. This led Dive et al. to design a new class of ACE2 inhibitors based on phosphinic peptide chemistry, which was successfully used to develop potent and highly selective ACE inhibitors [120]. These ACE2 inhibitors are pseudopeptide mimetics of peptide substrates in their transition states with a phosphinic zinc- binding group. Longer side-chains were introduced in the P_1_′ position to ensure potency towards ACE2 and this led to the selection of compounds with a P_1_′ phenyl-substituted isoxazole ring. A good fit between the P_1_ pseudoproline and the S_1_ subsite is partly responsible for the potency and selectivity of the inhibitor. In ACE2 the S_1_ Tyr^510^ is substituted with a smaller valine in ACE and this likely explains the P_1_ specificity. Finally, a histidine in the P_2_ position maintains the high potency towards ACE2 while reducing the inhibition of carboxypeptidase A. Compound 41FII (3-((1-[2-Acetylamino-3-(1H-imidazol-4-yl)-propionyl]-pyrrolidin-2-yl)-hydroxy-phosphinoyl)-2-(3-phenyl-isoxazol-5-ylmethyl)-propionic acid) had a *K*_i_ value of 0.4 nM and a 2600-fold selectivity over carboxypeptidase A, and may be a good candidate for defining biological substrates of ACE2 and its *in vivo* functions.

### Activation of ACE2

Despite the extensive work that has been carried out on the development of ACE2 inhibitors, it is likely that pharmacological activation of ACE2 is more beneficial for balancing the adverse effects of Ang II in cardiovascular disease. Prada et al. screened small drug-like molecules *in silico* by molecular docking into binding sites identified by the analysis of the differences between the molecular surfaces of the open and closed ACE2 conformations [121]. Xanthenone (XNT) and resorcinolnaphthalein, two of the highest scoring compounds directed at ACE2 structural sites, increased enzyme activity by 2-fold, but had no significant effect on ACE activity. Because of XNT’s superior solubility and toxicology profiles, its effects on cardiovascular physiology were tested *in vivo*. Chronic administration of XNT resulted in a decrease in blood pressure and improvements in cardiac function in spontaneously hypertensive rats (SHRs). Additionally, myocardial and renal fibrosis was reversed in the SHR and, in more recent studies, XNT improved endothelial function of diabetic and hypertensive rat vessels by decreasing oxidative stress [122].

Using a similar approach to that of Prada et al., a library of Food and Drug Administration (FDA)-approved drugs was screened using the apo form of human ACE2. The compounds targeted to the hinge region of ACE2 had the most pronounced effect on enzyme activity. The antihistamine hydroxyzine, the antipsychotic drug minithixen, and the antiprotozoan therapeutic diminazene aceturate (DIZE) had a significant effect on enzyme efficiency (*V*_max_/*K*_m_), with DIZE having the largest effect (two-fold). Minithixen and hydroxyzine increased the specificity for the substrate, whereas DIZE produced a large increase in *V*_max_ (four-fold) but a slight decrease in affinity. Interestingly, the titration of ACE2 with DIZE results in a biphasic dose–response curve with enzyme activation at low concentrations and partial inhibition at high concentrations with an *IC_50_* of 200 µM. DIZE exerts beneficial effect in various cardiovascular disease models including hypertension, type-1 diabetes, atherosclerosis [123] and Alzheimer’s disease [124]. However, in these studies the increased hydrolysis of Ang II *in vivo* was not confirmed, and so the precise mechanism of DIZE and XNT remain unclear. DIZE increases mRNA expression of ACE2 [125,126], so the up-regulation of ACE2 activity by compounds like DIZE and XNT could involve increased activation and/or gene expression.

### Recombinant ACE2 protein and peptides

Recently, a potential therapeutic role for recombinant human ACE2 (rhACE2) in pulmonary arterial hypertension (PAH) was investigated in a Phase IIa clinical trial [127]. There was a significant change in the Ang II/Ang-(1-7) in plasma of PAH patients, indicating a decrease in ACE2 activity. Treatment with rhACE2 was associated with a decrease in plasma markers for inflammation, increased levels of superoxide dismutase 2 (a marker of Mas I activation), and improved pulmonary haemodynamics. In addition, a reduction in plasma nitrotyrosine levels was observed at 4 and 24 h after rhACE2 administration, suggesting a decrease in total body oxidant stress.

The 740-amino acid soluble form of ACE2 showed good tolerability and safety in a first-in-man pharmacokinetics study [128]. Ang II levels decreased post-infusion of rhACE2 and Ang-(1-5) increased for all doses. However, there were no adverse cardiovascular effects observed suggesting that mechanisms exist to compensate for the changes in angiotensin peptide levels. rhACE has probably gained the most traction as a therapeutic intervention in the treatment of acute lung injury in patients with acute respiratory distress syndrome (ARDS) [129], and more recently in the treatment of COVID-19, for which it is currently in Phase II trials in Austria, Germany and Denmark (NCT04335136). These trials will be discussed in more detail in a review in this special ACE2 edition by Josef Penninger, who has pioneered the use of rhACE2 in ARDS.

While soluble rhACE2 can act as a decoy for the SARS-CoV-2, preventing viral infection, fragments of the N-terminal region of ACE2 or the SARS-CoV RBD have therapeutic potential for the treatment of COVID-19. Screening of peptide libraries identified a hexapeptide of the RBD that bound ACE2 with a *K*_D_ = 46 µM. Small molecules that inhibit SARS-CoV entry have also been identified by screening a chemical library of pharmacologically active compounds [130]. An oxazole-carboxamide derivative, SSAA09E2, blocks the binding of the SARS-CoV spike protein RBD to ACE2 but does not inhibit the enzymatic activity of ACE2.

Finally, using docking and molecular dynamics Baig et al. identified a peptide inhibitor that interferes with the interaction of ACE2 and the SARS-CoV-2 spike protein [131]. Alanine scanning was used to identify the 18- amino acid peptide (Phe^28^–Leu^45^) of the α1 helix of the ACE2 peptidase domain and binding energies and conformational stability were used to evaluate each residue for functional significance and structural stability. An overlapping 23-mer peptide (Ile^21^–Ser^43^) was synthesised by Zhang et al. and evaluated for binding to the spike RBD [132]. Bio-layer interferometry showed that the 23-mer associates with micromolar affinity to insect cell-derived RBD, but not to the human embryonic kidney (HEK) cell-expressed form. This suggests that post-translational modifications of the RBD, such as glycosylation, could play an important role in modulating peptide interactions with the SARS-CoV-2 RBD.

## Conclusion

Extensive progress has been made on the structural biology of ACE over the last two decades. This has been fuelled by the recent COVID-19 pandemic and the urgency to understand the molecular basis for host–viral protein interactions. High resolution crystal and cryo-EM structures solved at an unprecedented rate have determined that key residues in the N-terminal exosite of ACE2 are critical for binding of the host receptor to the spike protein of the virus. The higher affinity of ACE2 for the SARS-CoV-2 than for the SARS-CoV RBD, and its wide tissue distribution including the proximal airway, might partly explain the rapid transmission and severity of the disease.

Glycosylation has been an important consideration in the crystal structure determination of ACE proteins. ACE and ACE2 are decorated by heterogeneous *N*-linked glycans whose site occupancy has been modified to optimise crystallisation conditions. In the course of writing this review, deep mutagenesis of ACE2 revealed that the mutation of the Asn^90^ glycosylation site was highly favourable for RBD binding, and that the glycan at this position was predicted to obstruct ACE2 interaction. Glycan profiling will be an important part of future studies to understand this interaction and develop therapeutic interventions that disrupt RBD binding to ACE2 [113].

The hinge-bending motion of ACE and ACE2 are central to the mechanism by which they catalyse hydrolysis of peptide substrates where the movement of the two subdomains bring the catalytic components of the active site into a functional orientation. The recent ‘open’ crystal structure of the N-domain of ACE exposes a wide groove that permits substrates to access the active site. This groove in ACE and ACE2 likely interacts with substrates positioning them for different types of cleavage. ACE crystal structures have provided important insight into understanding substrate specificity and allowed for the design of increasingly useful compounds for the treatment of a range of human diseases.

## Open Access

Open access for this article was enabled by the participation of University of Bath in an all-inclusive *Read & Publish* pilot with Portland Press and the Biochemical Society under a transformative agreement with JISC.

## Abbreviations

Aβ: amyloid β
ACE: angiotensin converting enzyme
AcSDKP: acetyl-Ser-Asp-Lys-Pro
AnCE: *Drosophila* angiotensin converting enzyme
*Ano*ACE: *Anopheles* angiotensin converting enzyme
ARDS: acute respiratory distress syndrome
BPP: bradykinin-potentiating peptide
CoV: coronavirus
COVID-19: coronavirus infectious disease 2019
DIZE: diminazene aceturate
GPI: glycosylphosphatidylinositol
GnRH: gonadotropin-releasing hormone
*Lm*ACE: *Locusta migratoria* angiotensin converting enzyme
NPS: natriuretic peptide system
PAH: pulmonary arterial hypertension
RAAS: renin–angiotensin aldosterone system
RBD: receptor-binding domain
RBM: receptor-binding motif
rhACE2: recombinant human ACE2
RMSD: root mean square deviation
sACE: somatic ACE
SARS: severe acute respiratory syndrome
SHR: spontaneously hypertensive rat
tACE: testis ACE
XNT: Xanthenone
